# Increased glucocorticoid metabolism in diabetic kidney disease

**DOI:** 10.1371/journal.pone.0269920

**Published:** 2022-06-24

**Authors:** Daniel Ackermann, Bruno Vogt, Murielle Bochud, Michel Burnier, Pierre-Yves Martin, Fred Paccaud, Georg Ehret, Idris Guessous, Belen Ponte, Menno Pruijm, Antoinette Pechère-Bertschi, Heidi Jamin, Rahel Klossner, Bernhard Dick, Markus G. Mohaupt, Carine Gennari-Moser

**Affiliations:** 1 Department of Nephrology and Hypertension, University of Bern, Berne, Switzerland; 2 Swiss Kidney Project on Genes in Hypertension (SKIPOGH) Team, Lausanne, Switzerland; 3 Department for BioMedical Research, University of Bern, Berne, Switzerland; 4 Department of Internal Medicine, Sonnenhof, Lindenhofgruppe, Berne, Switzerland; 5 School of Medicine, University of Nottingham, Division of Child Health, Obstetrics & Gynaecology, Nottingham, United Kingdom; Universidad Nacional Autonoma de Mexico, MEXICO

## Abstract

**Aims:**

Glomerular damage indicated by proteinuria is a main symptom in diabetic nephropathy. Mineralocorticoid receptor (MR) antagonists (MRAs) are beneficial irrespective of aldosterone availability. Thus, we hypothesized an alternatively activated MR to promote glomerular damage in proteinuric diabetic nephropathy. Specifically, we aimed first to demonstrate the presence of steroid hormones serving as alternative MR targets in type II diabetic patients with proteinuric kidney disease, second whether MR selectivity was modified, third to characterize MR and glucocorticoid receptor (GR) expression and activity in glomerular cell types exposed to eu- and hyperglycemic conditions, fourth to characterize the pro-fibrotic potential of primary human renal mesangial cells (HRMC) upon stimulation with aldosterone and cortisol, and fifth to specify the involvement of the MR and/or GR in pro-fibrotic signaling.

**Materials and methods:**

Urinary steroid hormone profiles of patients with diabetic kidney disease were analyzed by gas chromatography–mass spectrometry and compared to an age and gender matched healthy control group taken out of a population study. In both cohorts, the activity of the MR pre-receptor enzyme 11β-hydroxysteroid dehydrogenase type 2 (HSD11B2), which inactivates cortisol to prevent it from binding to the MR, was assessed to define a change in MR selectivity. Expression of HSD11B2, MR and GR was quantified in HRMC and primary human renal glomerular endothelial cells (HRGEC). Activity of MR and GR was explored in HRMC by measuring the MR/GR down-stream signal SGK1 and the pro-fibrotic genes TGFB1, FN1 and COL1A1 in normal and high glucose conditions with the MR/GR agonists aldosterone/cortisol and the MR/GR antagonists spironolactone/RU486.

**Results:**

Patients with diabetic kidney disease excreted more tetrahydroaldosterone than the control group reaching significance in men. The excretion of MR-agonistic steroid hormones was only increased for 18-hydroxytetrahydrocorticosterone in diabetic women. The excretion of most glucocorticoids was higher in the diabetic cohort. Higher apparent systemic HSD11B2 activity suggested less activation of the MR by cortisol in diabetic patients. Both cell types, HRMC and HRGEC, lacked expression of HSD11B2. Hyperglycemic conditions did not change MR and GR expression and activity. Stimulation with both aldosterone and cortisol promoted upregulation of pro-fibrotic genes in HRMC. This effect of MR and/or GR activation was more pronounced in high glucose conditions and partially inhibited by MRAs and GR antagonists.

**Conclusions:**

In patients with diabetic kidney disease alternative MR activation is conceivable as cortisol and cortisone metabolites are increased. Systemic availability of active metabolites is counteracted via an increased HSD11B2 activity. As this cortisol deactivation is absent in HRMC and HRGEC, cortisol binding to the MR is enabled. Both, cortisol and aldosterone stimulation led to an increased expression of pro-fibrotic genes in HRMC. This mechanism was related to the MR as well as the GR and more marked in high glucose conditions linking the benefit of MRAs in diabetic kidney disease to these findings.

## Introduction

Diabetic nephropathy is a chronic kidney disease that develops in diabetic patients. It is characterized by progressive fibrosis and a defective glomerular filtration barrier [1]. Though in some patients with type 2 diabetes plasma aldosterone (Aldo) levels are increased, paradoxically in a substantial number of individuals a low Aldo availability prevails, [2–7]. The mineralocorticoid receptor (MR) is targeted by Aldo as primary agonist and is widely expressed within the kidneys [8, 9]. Activation of the MR in kidney cells leads to pro-inflammatory and pro-fibrotic responses [10–13]. In detail, Aldo stimulates the expression of the pro-fibrotic molecules transforming growth factor-β1 (TGFB1), plasminogen activator inhibitor 1, endothelin 1, placental growth factor, connective tissue factor, osteopontin, and galectin-3 in proximal tubule cells. TGFB1 promotes fibrosis by stimulating cellular transformation of many cells to fibroblasts. Aldo is known to increase the expression of TGFB1 in the heart [14] and in mesangial cells [15]. These pro-inflammatory and pro-fibrotic responses are prevented by MR antagonism.

Both, steroidal and non-steroidal MR antagonists (MRAs), such as spironolactone, eplerenone and finerenone are beneficial in the treatment of chronic kidney disease and diabetic nephropathy [13, 16–21]. All three MRAs significantly reduce proteinuria in addition to RAAS inhibitors in chronic kidney disease and diabetic nephropathy [22–24]. As MR activation promotes cardiovascular inflammation and fibrosis itself [25–27], MRAs are important drugs in reducing these adverse events finally leading to increased morbidity and mortality [28–31]. The beneficial effects of MRAs are not coupled to the presence of Aldo alone, as also angiotensin II (Ang II) and cortisol can lead to MR activation. MRAs inhibit renal injury induced by cortisol and Ang II in Aldo-deficient rodents [32, 33], and in rodents and humans treated with ACE-inhibitors or angiotensin receptor 1 (AT1R) blockers [34, 35]. The observation that MR antagonism decreases inflammation and fibrosis during conditions of high salt intake, which is associated with suppression of circulating Aldo concentrations, suggests that other environmental conditions might activate the MR. Ang II promotes MR activation and cortisol may activate the MR in cells that lack the enzyme 11beta-Hydroxysteroid dehydrogenase type 2 (HSD11B2). Cardiomyocytes lack HSD11B2, allowing cortisol binding to the MR. Cortisol promotes adverse cardiovascular effects, cardiac inflammation and fibrosis and this effect can be blocked by spironolactone, but not by the GR inhibitor RU486, suggesting a MR dependent mechanism [36, 37]. Aldo additionally stimulates expression of pro-inflammatory and pro-fibrotic genes in macrophages, an effect prevented by MRAs [38] or MR deletion [39].

In conditions with high systemic or local Aldo levels, any impact of MRAs is well explained. Yet, as diabetic nephropathy is frequently complicated by low-renin states with low Aldo levels, the observed benefits are less intuitive [7].

We hypothesized a role of the MR to induce fibrosis and consequently proteinuria.

Specifically, we aimed **first,** to define the presence of steroid hormones serving as alternative MR targets in type II diabetic patients with proteinuric diabetic kidney disease. To assess this, the urinary steroid hormone profile of patients with diabetic kidney disease was compared to that of a healthy age and gender matched control group. **Second**, whether MR selectivity was modified by assessing systemic HSD11B2 activity in the cohorts and by measuring HSD11B2 expression in glomerular endothelial and mesangial cells. **Third,** to characterize MR/GR expression and activity in glomerular endothelial and mesangial cells exposed to eu- and hyperglycemic conditions. SGK1 expression was assessed as down-stream signal of MR/GR activation. **Fourth,** to characterize the pro-fibrotic potential of primary human renal mesangial cells (HRMC) upon stimulation with aldosterone and cortisol, and **fifth**, to specify MR and GR involvement in this pro-fibrotic signaling. Transforming growth factor beta 1 (TGFB1), fibronectin 1 (FN1) and collagen 1A1 (COL1A1) were investigated as pro-fibrotic signals while the MR inhibitor spironolactone and the GR inhibitor RU486 were used to specify MR and GR dependency.

## Materials and methods

### Material and cell lines

Cell culture material was from Techno Plastic Products AG (TPP), Trasadingen, Switzerland. Collagen I coated petridishes were from Corning (Milian, Nesselenbach, Switzerland), while Poly-L-lysine and fibronectin for cell ware coating were from Sciencell (Chemie Brunschwig, Basel, Switzerland).

The primary cells HRMC (Sciencell # 4200) and HRGEC (Sciencell # 4000) and their corresponding media MCM (Sciencell 4201) and ECM (Sciencell 1001) with the supplements (MsCGS Cat. No. 4252 and ECGS Cat. No. 1052), penicillin/streptomycin (P/S, Cat. No. 0503) and FBS (Cat. No. 0010 and Cat. No. 0025) were obtained from Sciencell (Chemie Brunschwig, Basel, Switzerland). HEK293 (human embryonic kidney cells) and JEG-3 (a human choriocarcinoma cell line) were from ATCC, and their corresponding media DMEM (Cat. # 41965) and McCoy’s (Cat. # 36600) respectively, as well as the FBS, P/S and sodium pyruvate were from Gibco.

D (+)—Glucose monohydrate was from Merck (Zug, Switzerland). All steroid standards for GC-MS were purchased from Steraloids, Inc. (Newport, RI, USA).

Assay on demand primers for MR (Hs01031809_m1 *NR3C2*), HSD11B2 (Hs00388669_m1), SGK1 (Hs00178612_m1), GR (Hs00353740_m1 *NR3C1*), and *Cyclophilin A* (4326316E) were from Applied Biosystems (Thermo Fisher Scientific, Reinach, Switzerland). Primer pairs for TGFB1 (5’-actactacgccaaggaggtcac-3’ and 5’-tgcttgaacttgtcatagatttcg-3’), FN1 (5’-gggagaataagctgtaccatcg-3’ and 5’-tccattaccaagacacacacact-3’) and COL1A1 (5’-ctggacctaaaggtgctgct-3’ and 5’-gctccagcctctccatctt-3’) were designed by means of the Universal ProbeLibrary Assay Design Center software from Roche. All primers were intron-spanning and were synthesized by Microsynth AG (Balgach, Switzerland). Universal ProbeLibrary hydrolysis probes were purchased from Sigma-Aldrich (Buchs, Switzerland).

GC-MS instrument Nr. GC 6890N, MS 5973N was from Agilent (La Jolla California, USA).

7500 Fast Real-time PCR system machine was from Applied Biosystems (Thermo Fisher Scientific, Reinach, Switzerland).

### Diabetic patients and healthy controls

The diabetic cohort consisted of patients (men n = 21, women n = 20) with type 2 diabetes who referred to our outpatient clinic of the Department of Nephrology, University Hospital of Bern, Switzerland for the evaluation and treatment of their arterial hypertension. All patients had clinical signs of diabetic nephropathy, as high (≥3.4 mg/mmol creatinine) or very high (≥ 33.9 mg/mmol creatinine) albuminuria in 24h urine samples. Exclusion criteria were an estimated GFR <60ml/min, evidence of renovascular disease, history of malignant hypertension, systolic BP >210 mm Hg, cerebrovascular accident in the previous 12 months or current transient ischemic attacks, myocardial infarction within the previous 12 months or history of heart failure, pregnancy and breast feeding. As part of our standard procedure all drugs (e.g. ACE-inhibitors, angiotensin receptor or renin blockers, spironolactone and diuretics) influencing the renin-angiotensin-aldosterone system (RAAS) were stopped at least 1 week prior to urinary steroid hormone analysis. Because of their vasodilating effect only doxazosin, clonidin and amlodipin were given to the patients during the period of steroid hormone analysis. Antihyperglycemic agents such as sulfonylureas (e.g. glimepiride, gliclazide), gliptins, dipeptidylpeptidase-4 inhibitors, thiazolidinediones (pioglitazone), metformin and insulin were continued during steroid hormone analysis. Clinical work up of diabetic patients was done according to standard protocols, 24h urine was collected and analysis was performed retrospectively. Detailed characteristics of both cohorts are summarized separate for men and women in Table 1.

**Table 1:** Characteristics diabetic patients-healthy controls.

Variables	Diab.	Controls	p-values
**Men**			
Age, years	56 (12)	57.5 (12.3)	0.66
BMI, kg/m^2^	31.7 (5.1)	27.2 (3.6)	<0.001
Systolic blood pressure, mmHg	148.1 (18.5)	126.5 (18)	<0.001
Diastolic blood pressure, mmHg	87.2 (15.8)	79.5 (9)	0.034
Fasting glucose, mmol/l	9 (2.56)	5.5 (0.9)	<0.001
Total cholesterol, mmol/l	4.42 (1.24)	5.4 (1.2)	0.004
LDL, mmol/L	2.86 (0.8)	3.4 (1)	0.05
eGFR, mL/min/1.73m^2^	73.3 (17)	88.5 (15.9)	0.001
24h urinary albumin, mg	482 (643)	12.3 (11)	0.003
Urinary albumin/creatinine mg/mmol	38 (59)	1.28 (1.3)	0.009
**Women**			
Age, years	63.7 (13)	60.5 (13.4)	0.37
BMI, kg/m2	35.3 (6.3)	25.1 (4.6)	<0.001
Systolic blood pressure, mmHg	153.7 (22.6)	126.1 (17.1)	<0.001
Diastolic blood pressure, mmHg	84.2 (11.7)	76.8 (9)	0.022
Fasting glucose, mmol/l	9.1 (3)	5.14 (0.5)	<0.001
Total cholesterol, mmol/l	4.86 (1.16)	5.24 (0.85)	0.22
LDL, mmol/L	2.84 (0.97)	3.16 (0.75)	0.2
eGFR, mL/min/1.73m2	72.8 (16.7)	86 (13.6)	0.006
24h urinary albumin, mg	302 (586)	11.6 (11)	0.004
Urinary albumin/creatinine mg/mmol	49 (92)	2.2 (2.9)	0.008

Body mass index (BMI), estimated glomerular filtration rate (eGFR)

Data are mean and standard deviation (SD) unless otherwise specified.

In order to compare diabetic patients to a matched control we chose healthy volunteers (men n = 155, women n = 161) from the Swiss Kidney Project on Genes in Hypertension (SKIPOGH) cohort. In SKIPOGH, participants with an endocrine disorder or endocrine disrupting drugs were excluded, but still had comparable renal function (eGFR >60 ml/min) [40], which makes them suitable as a control group. The SKIPOGH study is a multicentre Swiss study of the University clinics of Berne, Lausanne and Geneva. Briefly, healthy volunteers were recruited between 2009–2013 in these 3 cities as previously described [41]. The selected participants had no RAAS treatment, did not take any diuretics, had no renal diseases or diabetes. In this protocol, urinary samples of the control cohort were collected for day and night separately to differentiate between circadian blood pressure regulation. For the purpose of this study, day and night urine samples were combined to provide 24h excretion of urinary steroid hormones. To take potential incomplete urine collection into account, we excluded patients and participants with a 24h urine volume below 300ml and added urinary creatinine excretion per kilogram body weight as covariate in the analyses in both cohorts [42]. Urine aliquots were stored at -80°C until further analysis. Analysis was done retrospectively.

Sampling was done prospectively. Age and gender matching to the diabetic cohort was done in a case-control study, retrospectively. Matching details: age group 1932–1991 was matched 1–6:1–6 (number of diabetic patients to number of controls).

All parts of the studies were approved by the ethics committee of the Canton of Berne, Vaud or Geneva as required for the sample collection according to the Declaration of Helsinki. All patients and participants were only included in the study after signing informed consent.

### Gas chromatography–mass spectrometry (GC-MS)

Urinary steroid hormones were analyzed by gas chromatography-mass spectrometry (GC-MS) according to the method originally described by Shackleton [43] and applied by us as previously reported [44]. The volume of 1.5ml of 24h urines was used for analysis. Sample preparation consisted of pre-extraction, enzymatic hydrolysis, extraction from the hydrolysis mixture, derivatization, and gel filtration. Medroxyprogesterone (2.5 μg) was added as recovery standard to 1.5 mL urine. The sample was extracted on a Sep Pak C18 column (Waters Corp., Milford, MA, USA), dried, reconstituted in 0.1 mol/L acetate buffer (pH 4.6), and hydrolyzed with powdered Helix pomatia enzyme (12.5 mg) and 12.5 μL of β-glucuronidase/arylsulfatase liquid enzyme at 55°C for 3 hours. The resulting free steroids were extracted on a Sep Pak C18 cartridge and a standard mixture (5α-androstane-3α-17α-diol, stigamsterol, and cholesteryl butyrate, 2.5 μg of each, and 0.15 μg of 3β5β-THAldo) was added. The sample was derivatized to form the methyloxime-trimethylsilyl ethers, and gel filtration on a Lipidex 5000 column (Perkin Elmer Corp., Wellesley, MA, USA) was added. Samples were analyzed on a Hewlett Packard gas chromatograph 6890 (Palo Alto, CA, USA) equipped with the mass selective detector 5973 and the auto injector 7683 by selective ion monitoring for each measured compound against a known calibration standard (3β5β-THAldo against 3α5β-THAldo and all other steroids against stigmasterol). Ions were chosen with respect to their retention time during a temperature-programmed 35-minute run (210–265°C). Software used was MassHunter (Agilent, USA).

The reproducibility of the applied GC-MS is continuously monitored by internal quality control and the steroid hormone laboratory participates monthly in an external quality control organized by the Foundation for Quality Medical Laboratory Diagnostics skml (Stitching Kwaliteitsbewaking Medische Laboratoriumdiagnostiek, Nijmegen, The Netherlands). We focused on mineralocorticoid (i.e. tetrahydroaldosterone (TH-Aldo), tetrahydrodeoxycorticosterone (TH-DOC), tetrahydrocorticosterone (THB) and 18-hydroxytetrahydrocorticosterone (18-OH-THA)) and glucocorticoid metabolites (tetrahydrosubstance S (THS), cortisol (F), 5α-tetrahydrocortisol (5α-THF), 5β-tetrahydrocortisol (5β-THF), α-cortol, β-cortol, cortisone (E), tetrahydrocortisone (THE), α-cortolone and β-cortolone. The apparent enzyme activities of Aldo synthase (CYP11B2), steroid 11β-hydroxylase (CYP11B1), 11β-hydroxysteroid dehydrogenase type 2 (HSD11B2) and whole body 11β-hydroxysteroid dehydrogenase (HSD11B) as the sum of HSD11B1 and HSD11B2 were calculated. The activity of the Aldo synthase (CYP11B2) was calculated as ratio of THB/TH-Aldo and as ratio of 18-OH-THA/TH-Aldo. CYP11B1 enzyme activity was calculated as ratio of THS/cortisol. Whole body HSD11B and HSD11B2 activity were calculated as the urinary ratio of (5β-THF+5α-THF)/THE and as cortisol/cortisone respectively. A high ratio reflects a lower activity and a low ratio indicates a higher activity.

Steroid hormone concentrations are displayed in ug/24h and are shown as dot blot with Log (10) scale in the figures and with absolute values ± SEM in Table 2. The enzyme activites are shown as dot blot ratios in the figures and with absolute values ± SEM in Table 3.

**Table 2:** Absolute values of the urinary steroid hormone metabolites of the two human cohorts.

Mineralocorticoid metabolites	ug/24h	p-value
	mean +/- SEM, n	
TH-Aldo diabetic men	35.69 ± 6.338, n = 21	0.031
TH-Aldo control men	23.01 ± 1.233, n = 155	
TH-Aldo diabetic women	35.92 ± 7.428, n = 20	0.391
TH-Aldo control women	23.14 ± 1.529, n = 161	
18-OH-THA diabetic men	78.2 ± 15.04, n = 19	0.391
18-OH-THA control men	69.2 ± 9.072, n = 154	
18-OH-THA diabetic women	119.7 ± 19.71, n = 17	<0.0001
18-OH-THA control women	56.15 ± 8.236, n = 159	
THB diabetic men	134.5 ± 14.65, n = 21	0.484
THB control men	159.5 ± 6.44, n = 155	
THB diabetic women	142 ± 22.47, n = 20	0.578
THB control women	113.3 ± 7.303, n = 161	
TH-DOC diabetic men	6.395 ± 0.9297, n = 21	0.289
TH-DOC control men	7.284 ± 0.4031, n = 155	
TH-DOC diabetic women	8.24 ± 2.714, n = 20	0.979
TH-DOC control women	6.848 ± 0.6151, n = 161	
**Glucocorticoid metabolites**		
THS diabetic men	94.68 ± 9.324, n = 21	0.006
THS control men	65.58 ± 2.706, n = 155	
THS diabetic women	74.83 ± 7.069, n = 20	0.006
THS control women	51.13 ± 2.646, n = 161	
5α-THF diabetic men	2047 ± 194.8, n = 21	0.002
5α-THF control men	1390 ± 59.78, n = 154	
5α-THF diabetic women	1614 ± 446.9, n = 20	0.0004
5α-THF control women	627.8 ± 36.66, n = 159	
5β-THF diabetic men	2500 ± 269.4, n = 21	0.013
5β-THF control men	1695 ± 61.85, n = 154	
5β-THF diabetic women	2302 ± 258, n = 20	<0.0001
5β-THF control women	1164 ± 58.92, n = 159	
α-cortol diabetic men	455 ± 39.45, n = 21	0.013
α-cortol control men	316.7 ± 10.17, n = 155	
α-cortol diabetic women	487.8 ± 73.75, n = 20	<0.0001
α-cortol control women	216.3 ± 9.463, n = 161	
cortisone diabetic men	182.8 ± 18.4, n = 21	0.402
cortisone control men	173.8 ± 6.047, n = 155	
cortisone diabetic women	229.5 ± 34.34, n = 20	0.009
cortisone control women	144.5 ± 8.794, n = 161	
cortisol diabetic men	132.1 ± 21.42, n = 21	0.013
cortisol control men	156.9 ± 6.009, n = 155	
cortisol diabetic women	161 ± 27.08, n = 20	0.471
cortisol control women	126.6 ± 7.409, n = 161	
β-cortol diabetic men	511.3 ± 51.29, n = 21	0.769
β-cortol control men	509 ± 20.37, n = 155	
β-cortol diabetic women	651.6 ± 91.68, n = 20	<0.0001
β-cortol control women	301.7 ± 16.81, n = 161	
THE diabetic men	4507 ± 536.6, n = 21	0.004
THE control men	2885 ± 110.9, n = 151	
THE diabetic women	3603 ± 420.0, n = 20	<0.0001
THE control women	1953± 139.7, n = 159	
α-cortolone diabetic men	1592 ± 147.4, n = 20	0.009
α-cortolone control men	1120 ± 43.83, n = 153	
α-cortolone diabetic women	1538 ± 164.1, n = 20	<0.0001
α-cortolone control women	783.5 ±32.76, n = 158	
β-cortolone diabetic men	755.9 ± 51.42, n = 20	0.113
β-cortolone control men	644.2 ± 22.62, n = 154	
β-cortolone diabetic women	811.9 ±80.59, n = 20	<0.0001
β-cortolone control women	359.9 ±17.44, n = 161	

**Table 3:** Absolute values of the calculated enzymatic activities of the two human cohorts.

Calculated enzymatic activities	mean +/- SEM, n	p-value
CYP11B2 (THB/TH-Aldo) diabetic men	6.479 ± 1.299, n = 21	0.027
CYP11B2 (THB/TH-Aldo) control men	8.882 ± 0.5929, n = 155	
CYP11B2 (THB/TH-Aldo) diabetic women	6.241 ± 1.042, n = 20	0.834
CYP11B2 (THB/TH-Aldo) control women	6.411 ± 0.3256, n = 161	
CYP11B2 (18-OH-THA/TH-Aldo) diabetic men	4.808 ± 1.545, n = 21	0.541
CYP11B2 (18-OH-THA/TH-Aldo) control men	3.867 ± 0.4918, n = 155	
CYP11B2 (18-OH-THA/TH-Aldo) diabetic women	5.607 ± 1.395, n = 20	0.144
CYP11B2 (18-OH-THA/TH-Aldo) control women	3.317 ± 0.3976, n = 161	
CYP11B1 (THS/cortisol) diabetic men	1.176 ± 0.298, n = 21	<0.0001
CYP11B1 (THS/cortisol) control men	0.4697 ± 0.02239, n = 155	
CYP11B1 (THS/cortisol) diabetic women	0.6595 ± 0.08961, n = 20	0.0595
CYP11B1 (THS/cortisol) control women	0.4829 ± 0.02434, n = 161	
HSD11B2 (F/E) diabetic men	0.7214 ± 0.08597, n = 21	0.0004
HSD11B2 (F/E) control men	0.9214 ± 0.02052, n = 155	
HSD11B2 (F/E) diabetic women	0.7795 ± 0.1299, n = 20	0.0002
HSD11B2 (F/E) control women	0.9215 ± 0.02232, n = 161	
Whole HSD11B (THF+5α-THF)/THE diabetic men	1.119 ± 0.0709, n = 21	0.1504
Whole HSD11B (THF+5α-THF)/THE control men	1.663± 0.1720, n = 151	
Whole HSD11B (THF+5α-THF)/THE diabetic women	1.141 ± 0.1458, n = 20	0.1396
Whole HSD11B (THF+5α-THF)/THE control women	2.071 ± 0.4288, n = 158	

### Cell culture

HRMC were cultured in Poly-L-lysine coated cell ware with MCM medium containing 2% FBS. MCM contained 500 ml basal medium, 10 ml fetal bovine serum, 5 ml MC growth supplement, and 5 ml penicillin/streptomycin (P/S) solution. HRMC were used up to passage 6.

HRGEC were cultured in fibronectin coated or collagen I coated cell ware using the medium ECM containing 5% FBS. ECM contained 500 ml basal medium, 25 ml fetal bovine serum, 5 ml EC growth supplement and 5 ml penicillin/streptomycin solution. HRGEC were used up to passage 6.

Basal glucose concentration of MCM and ECM medium was 5.55mM. For high glucose conditions 20mM glucose was added to the basal medium resulting in an end concentration of 25.55mM glucose.

HEK293 were cultured in DMEM containing P/S, sodium pyruvate and 10% FBS.

JEG-3 cells were cultured in McCoy’s containing P/S and 10% FBS.

### Real-time PCR

Primary HRMC and HRGEC in S3 Fig were cultured for 72h either in basal glucose conditions (5.55mM) or in high glucose conditions (25.55mM). Medium was renewed every 24h.

Primary HRMC and HRGEC in Fig 5 were cultured in basal glucose conditions as described above.

Primary HRMC and HRGEC in S4 Fig were cultured for 72h either in basal glucose conditions (5.55mM) or in high glucose conditions (25.55mM). Medium was renewed every 24h.

Primary HRMC in Fig 6A were cultured for 72h in basal glucose conditions (5.55mM), while HRMC in Fig 6B were cultured for 72h in high glucose conditions (25.55mM). Medium was renewed every 24h. After the 72h priming period, HRMC in Fig 6A and 6B were stimulated for additional 24h with aldosterone (10^-7^M) or cortisol (10^-7^M). EtOH was the solvent of the steroid hormones and is displayed in the baseline control.

Primary HRMC in Fig 7 were cultured for 72 in high glucose conditions (25.55mM). Medium was renewed every 24h. After the 72h priming period, HRMC were stimulated for additional 24h with aldosterone (10^-7^M) or cortisol (10^-7^M) and with the combination of aldosterone or cortisol with spironolactone (10^-6^M) and RU486 (10^-6^M) in steroid free medium (charcoal treated MCM) containing high glucose levels (25.55mM). EtOH was the solvent. It is displayed in the baseline control. The MR and GR inhibitors spironolactone and RU486 respectively were added 1h prior to the steroid hormones, in order to allow binding to the receptors before challenging the cells with the ligands.

Primary HRMC in S5 Fig were cultured for 72 in baseline glucose conditions (5.55mM). Medium was renewed every 24h. After the 72h priming period, HRMC were stimulated for additional 24h with aldosterone (10^-7^M) or cortisol (10^-7^M) in steroid free medium (charcoal treated MCM) containing normal glucose levels (5.55mM). EtOH was the solvent. It is displayed in the baseline control.

At the end of the incubation period extraction of total RNA was performed using the Trizol method. RNA was reverse transcribed (Prom II RT, Promega). Assay on demand primers were used for human MR (NR3C2), GR (NR3C1), HSD11B2, SGK1 and Cyclophilin A. For TGFB1, FN1 and COL1A1 primer pairs were designed as described above. Cyclophilin A served as endogenous control. HEK293 and JEG-3 were used as positive controls. Results are displayed as fold difference. Assays were performed in triplicates.

### Statistical methods

Characteristics of the two cohorts were normally distributed and compared with two sided t-test. The cohorts were properly matched for gender and age. To describe normal levels as accurate as possible, all available controls from the SKIPOGH cohort, who matched for gender and age, were included. Data of the steroid hormones were not normally distributed as assessed with the D’Agostino & Pearson normality test and the Shapiro-Wilk normality test. Data in figures are presented as median with interquartile range in a log (10) scale. Data in tables are presented as mean ± SEM. Statistical significance between steroid hormones was tested with the non-parametric, unpaired Kolmogorov-Smirnov test.

Cell culture data are presented as mean ± SD. One-way ANOVA with Dunnett’s multiple comparisons test was used to compare more than two parameters with each other. To compare two conditions with each other unpaired t test was used.

Significance was assigned at p<0.05. *** p<0.0001, ** p<0.01. * p<0.05. All statistical analyses were performed using GraphPad PRISM version 7 (PRISM, USA).

## Results

### Urinary excretion of steroid hormones in diabetic patients and healthy controls

Phenotypic characteristics of diabetic patients and healthy controls are shown in Table 1. Mean age of diabetic patients and their controls was 60 and 59 years, respectively. Diabetic patients had a higher BMI, higher systolic blood pressure and fasting glucose. Urinary metabolites and the calculated enzymatic activities are shown separately for men and women. In the diabetic cohort 21 men and 20 women where included while 155 men and 161 women matched as healthy controls. Because of the clinical setting there was no missing data in the diabetic cohort.

**Fig 1:**
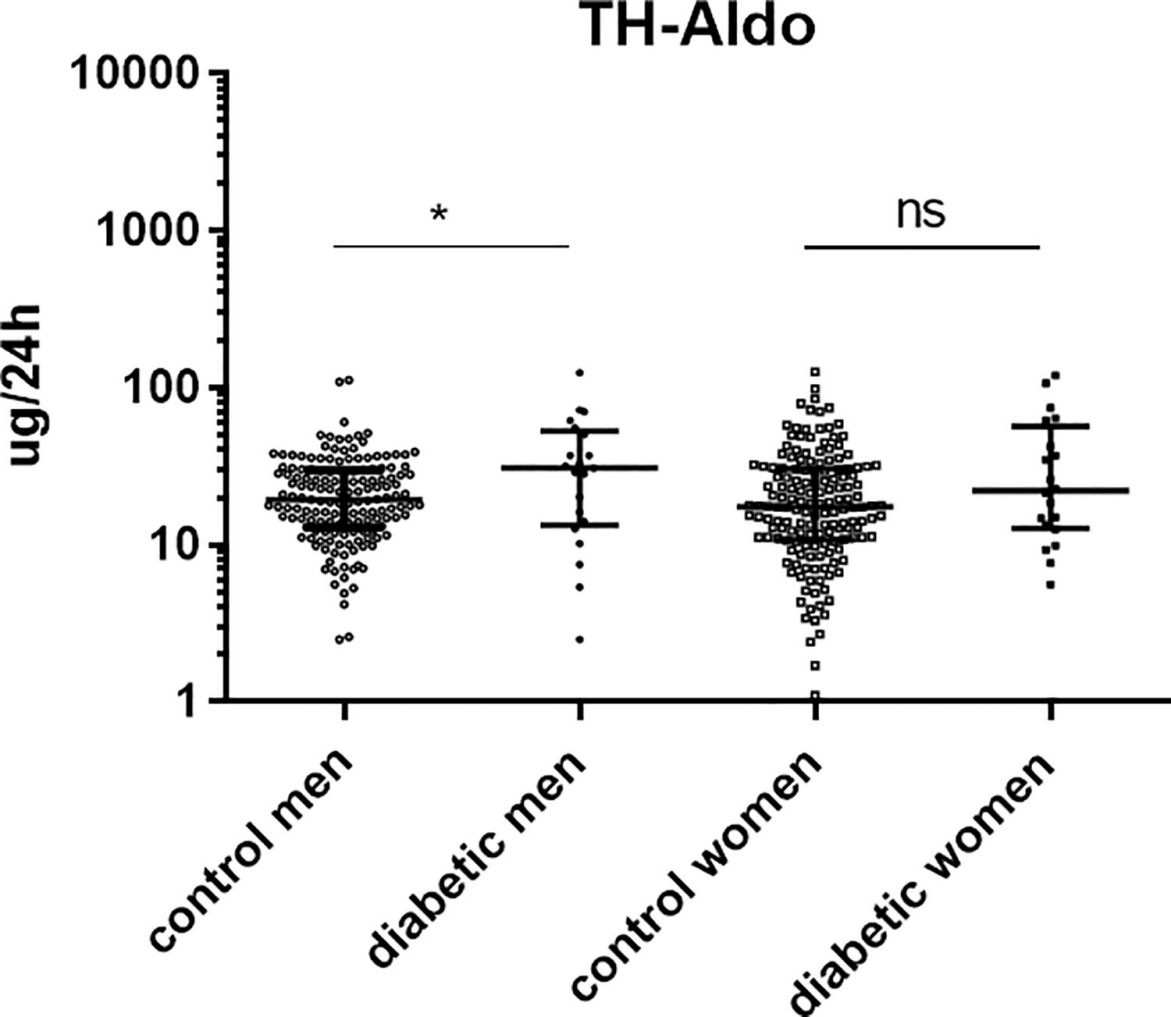
Urinary excretion of tetrahydroaldosterone (TH-Aldo) in diabetic patients and healthy controls. Urinary excretion of the mineralocorticoid metabolite TH-Aldo in healthy controls (n = 155 men, n = 161 women) and diabetic patients (n = 21 men, n = 20 women) measured by GC-MS. The groups were matched for age and gender. Diabetic men significantly excreted more TH-Aldo then their controls, while this finding was not significant in women. TH-Aldo *p = 0.031 (men), ns = not significant (women). Kolmogorov-Smirnov test (non-parametric, unpaired). Dot blot is shown with Log (10) scale and steroid concentrations are displayed in ug/24h. White circles = control men, black circles = diabetic men, white squares = control women, black squares = diabetic women. *p<0.05, **p<0.01, ***p<0.0001, ns = not significant.

Diabetic men and women showed a higher urinary excretion of TH-Aldo than their non-diabetic controls; however, this finding was statistically only significant in men (Fig 1, Table 2). Urinary excretions of TH-DOC, THB and 18-OH-THA were similar except the higher excretion of 18-OH-THA in diabetic women (S1 Fig, Table 2). The estimated activity of the Aldo synthase (CYP11B2) was calculated with 2 ratios: a) THB/TH-Aldo and b) 18-OH-THA/TH-Aldo. A high ratio reflects a lower activity and a low ratio indicates a higher activity. The CYP11B2 activity (calculated with THB/TH-Aldo) was significantly increased in diabetic men (Table 3), which could explain a higher urinary excretion of TH-Aldo in diabetic men. With 18-OH-THA/TH-Aldo no difference was found in men (Table 3). CYP11B2 activity of diabetic women seemed unchanged (Table 3). An overview of metabolism of mineralocorticoids is provided in S1 Fig.

**Fig 2:**
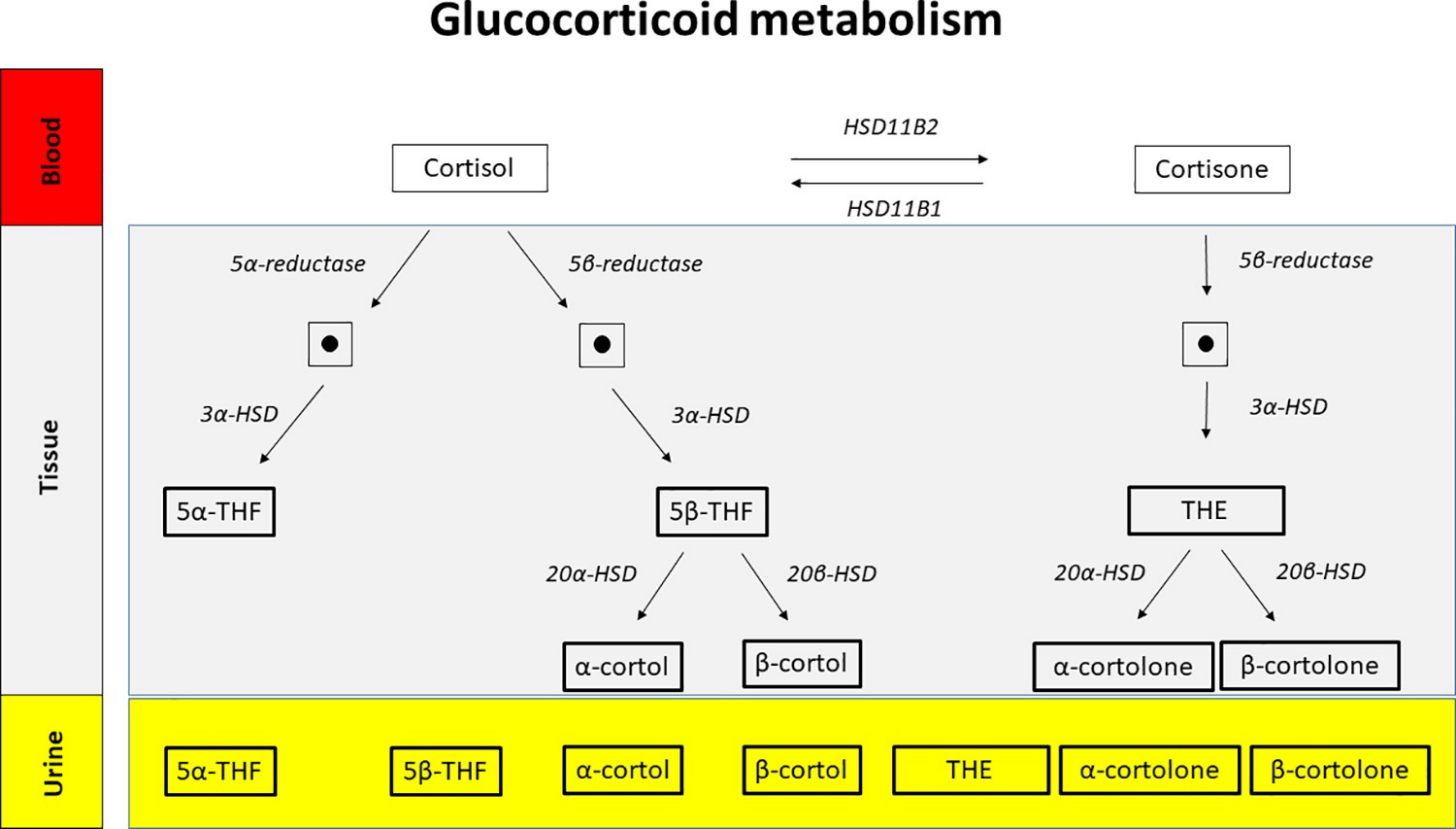
Glucocorticoid metabolism. Glucocorticoid metabolites in blood, tissue and urine of humans. Bulky framed urinary steroid hormone metabolites were measured in urine by GC-MS and found to be increased in diabetic patients as compared to a healthy control group. Many more glucocorticoid metabolites exist. For convenience, only the metabolites discussed in this paper are shown. The dots stand for intermediates that are not discussed in the manuscript. *p<0.05, **p<0.01, ***p<0.0001, ns = not significant.

**Fig 3:**
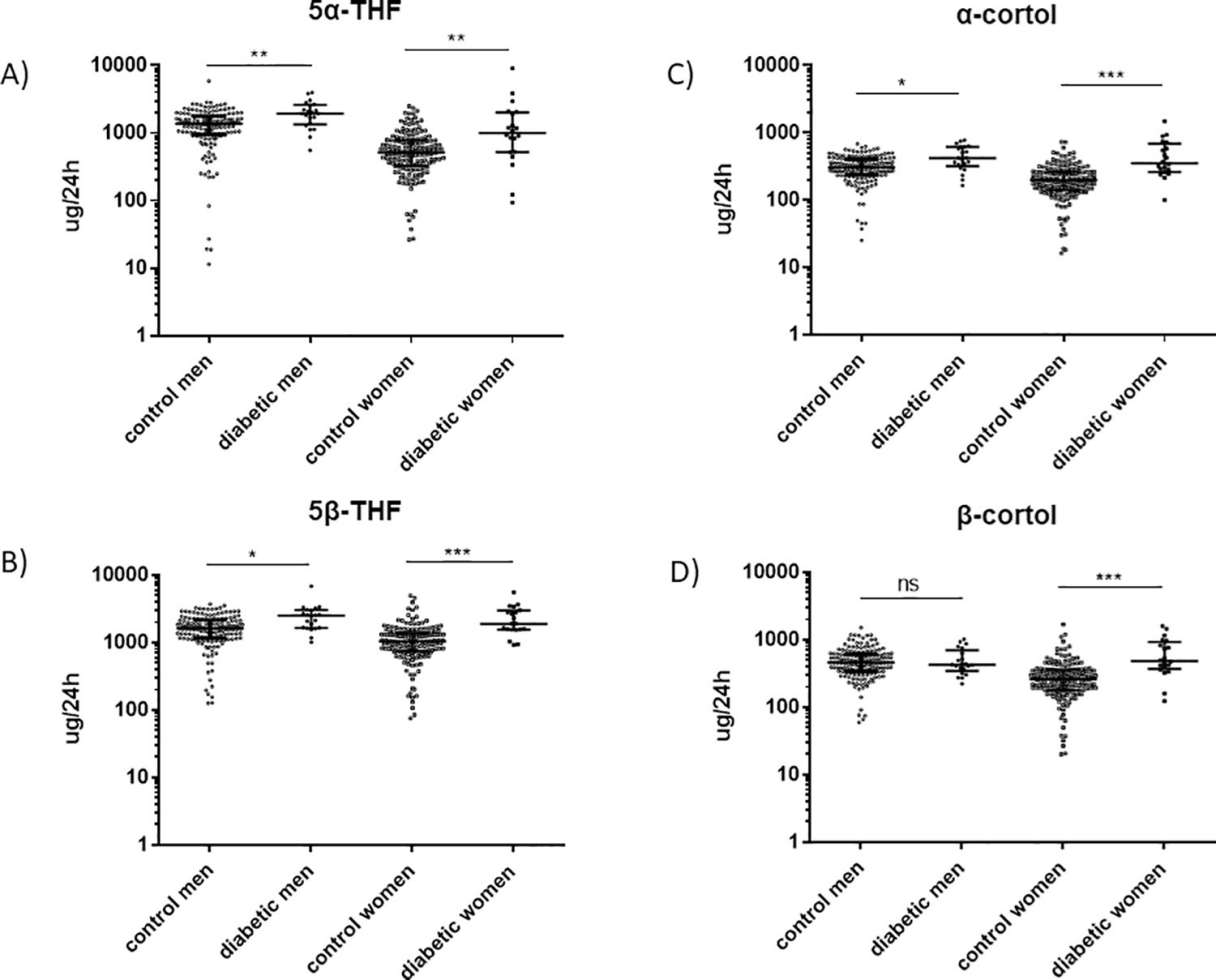
Urinary excretion of 5α-tetrahydrocortisol (5α-THF), 5β-tetrahydrocortisol (5β-THF), α-cortol and β-cortol in diabetic patients and healthy controls. Urinary excretion of the cortisol metabolites 5α-THF, 5β-THF, α-cortol and β-cortol in non-diabetic (n = 155 for men, n = 161 for women) and diabetic patients (n = 21 for men and n = 20 for women) measured by GC-MS. The groups were matched for age and gender. Diabetic men excreted significantly more 5α-THF, 5β-THF and α-cortol as compared to control men. β-cortol was not significantly changed between diabetic and non-diabetic men. Diabetic women excreted significantly more 5α-THF, 5β-THF, α-cortol and β-cortol as compared to control women. A) 5α-THF **p = 0.002 (men), **p = 0.0004 (women). B) 5β-THF *p = 0.013 (men), ***p<0.0001 (women). C) α-cortol *p = 0.013 (men), ***p<0.0001 (women). D) β-cortol ns (men), ***p<0.0001 (women). Kolmogorov-Smirnov test (non-parametric, unpaired). Dot blot is shown with Log (10) scale and steroid concentrations are displayed in ug/24h. White circles = control men, black circles = diabetic men, white squares = control women, black squares = diabetic women. *p<0.05, **p<0.01, ***p<0.0001, ns = not significant.

**Fig 4:**
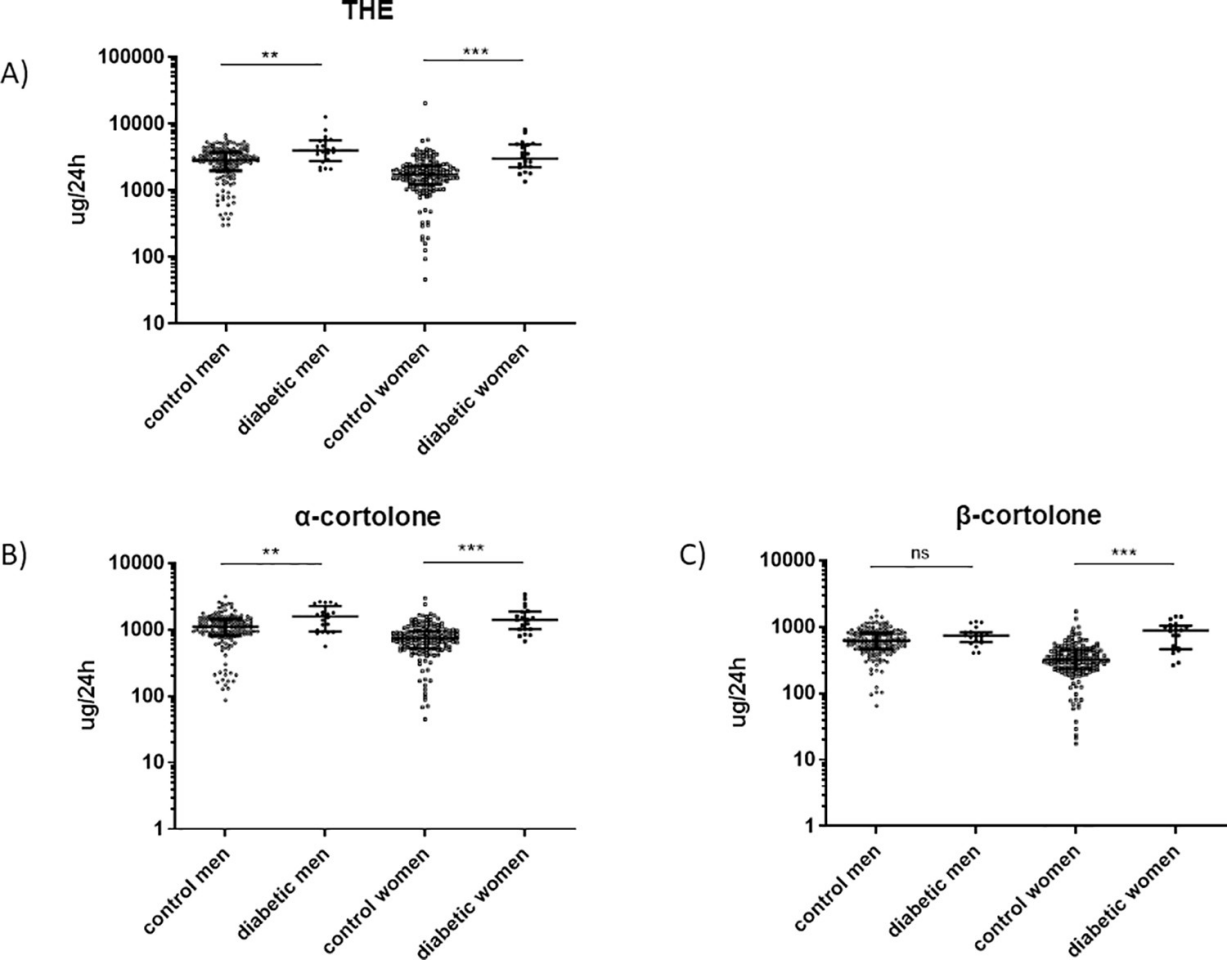
Urinary excretion of tetrahydrocortisone (THE), α-cortolone and β-cortolone in diabetic patients and healthy controls. Urinary excretion of the cortisone metabolites THE, α-cortolone and β-cortolone in non-diabetic (n = 155 for men, n = 161 for women) and diabetic patients (n = 21 for men and n = 20 for women) measured by GC-MS. The groups were matched for age and gender. Diabetic women excreted significantly more THE, α-cortolone and β-cortolone as their healthy controls. Men excreted significantly more THE and α-cortolone as healthy controls. There was no significant change in β-cortolone excretion between diabetic and healthy men. A) THE **p = 0.004 (men), ***p<0.0001 (women). B) α-cortolone **p = 0.009 (men), ***p<0.0001 (women). C) β-cortolone ns (men), ***p<0.0001 (women). Kolmogorov-Smirnov test (non-parametric, unpaired). Dot blot is shown with Log (10) scale and steroid concentrations are displayed in ug/24h. White circles = control men, black circles = diabetic men, white squares = control women, black squares = diabetic women. *p<0.05, **p<0.01, ***p<0.0001, ns = not significant.

An overview of metabolism of glucocorticoids is shown in Fig 2. Both, diabetic men and women showed a higher excretion of cortisol (Fig 3) and cortisone (Fig 4) metabolites as compared to the healthy cohort. In detail, urinary excretion of the down-stream metabolites of cortisol (5α-THF and 5β-THF reflecting the major component of cortisol synthesis and, α-cortol and β-cortol) was significantly higher in diabetic men and women except β-cortol in men (Fig 3A–3D, Table 2). Urinary excretion of the down-stream metabolites of cortisone (THE, α-cortolone and β-cortolone) was significantly increased in diabetic patients except β-cortolone in men (Fig 4A–4C, Table 2).

Diabetic patients excreted also more THS (the urinary metabolite for 11-deoxycortisol) than the control groups (S2 Fig, Table 2). Cortisol excretion per se was significantly decreased in diabetic men, while there was no significant difference in cortisol excretion between diabetic and control women (S2 Fig, Table 2). Cortisone excretion per se was increased in diabetic women, while no difference was found in men (S2 Fig, Table 2).

Apparent CYP11B1 enzyme activity calculated as ratio of THS/cortisol was lower in diabetic men, whereas women did not show a significant difference in CYP11B1 activity (Table 3).

Whole body HSD11B activity, reflecting HSD11B1 and HSD11B2 activity together was not changed between groups (Table 3). However, diabetic men and women showed increased systemic HSD11B2 activity (Table 3).

#### mRNA expression of MR and GR in HRMC and HRGEC upon stimulation with glucose

HRMC and HRGEC were cultured as described above. RNA was isolated and real-time PCR was performed to detect mRNA levels of MR and GR. High glucose levels (25.55mM) did not change MR or GR expression in HRMC and HRGEC. ns = not significant. S3A–S3D Fig.

#### mRNA expression of HSD11B2 in HRMC and HRGEC

**Fig 5:**
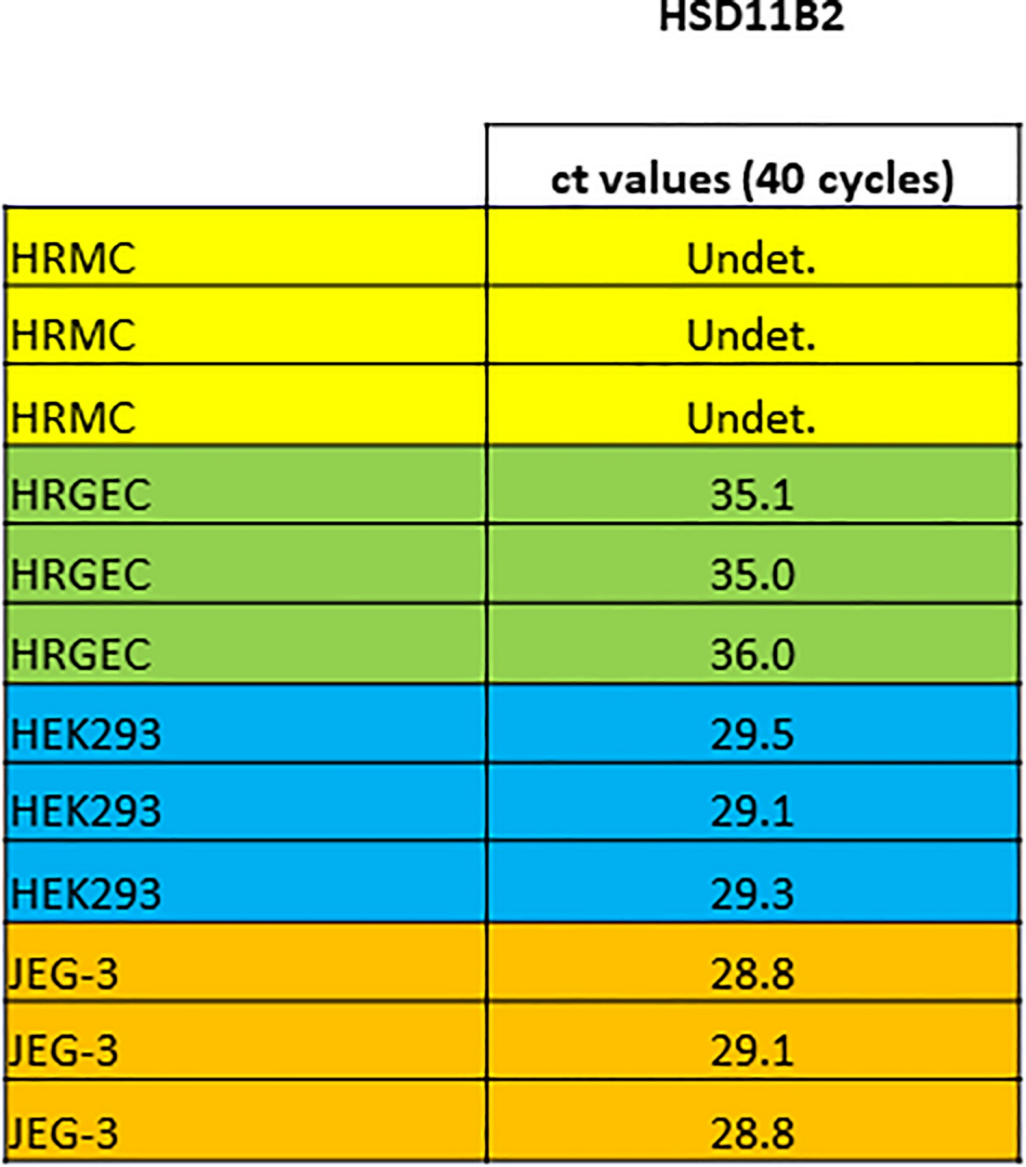
mRNA expression of HSD11B2 in HRMC and HRGEC. mRNA expression of the enzyme HSD11B2 was assessed by Real-time PCR. Amplification cycle number was 40. Ct values are shown. Gene detection was defined to be positive at ct values <35. HRMC: no detection of HSD11B2 (Undet. = undetected), ct >40. HRGEC: no detection of HSD11B2, ct > 35. HEK293: HSD11B2 detected, ct values ~ 29. JEG-3: HSD11B2 detected, ct values ~ 29. HEK293 and JEG-3 were used as positive controls. *p<0.05, **p<0.01, ***p<0.0001, ns = not significant.

HRMC, HRGEC, HEK293 and JEG-3 were cultured as described above. RNA was isolated and real-time PCR was performed to detect mRNA levels of the enzyme HSD11B2. No expression of HSD11B2 could be detected in HRMC and HRGEC (ct values > 35, 40 cycles). In the positive controls, HEK293 and JEG-3, HSD11B2 was expressed with ct values of 28–29. Fig 5.

#### mRNA expression of SGK1 in HRMC and HRGEC upon stimulation with glucose

HRMC and HRGEC were cultured as described above. RNA was isolated and real-time PCR was performed to detect mRNA levels of SGK1. High glucose levels did not change SGK1 expression levels in HRMC and HRGEC. ns = not significant (S4A and S4B Fig).

#### mRNA expression of SGK1 in HRMC stimulated with aldosterone and cortisol in normal and high glucose conditions

**Fig 6:**
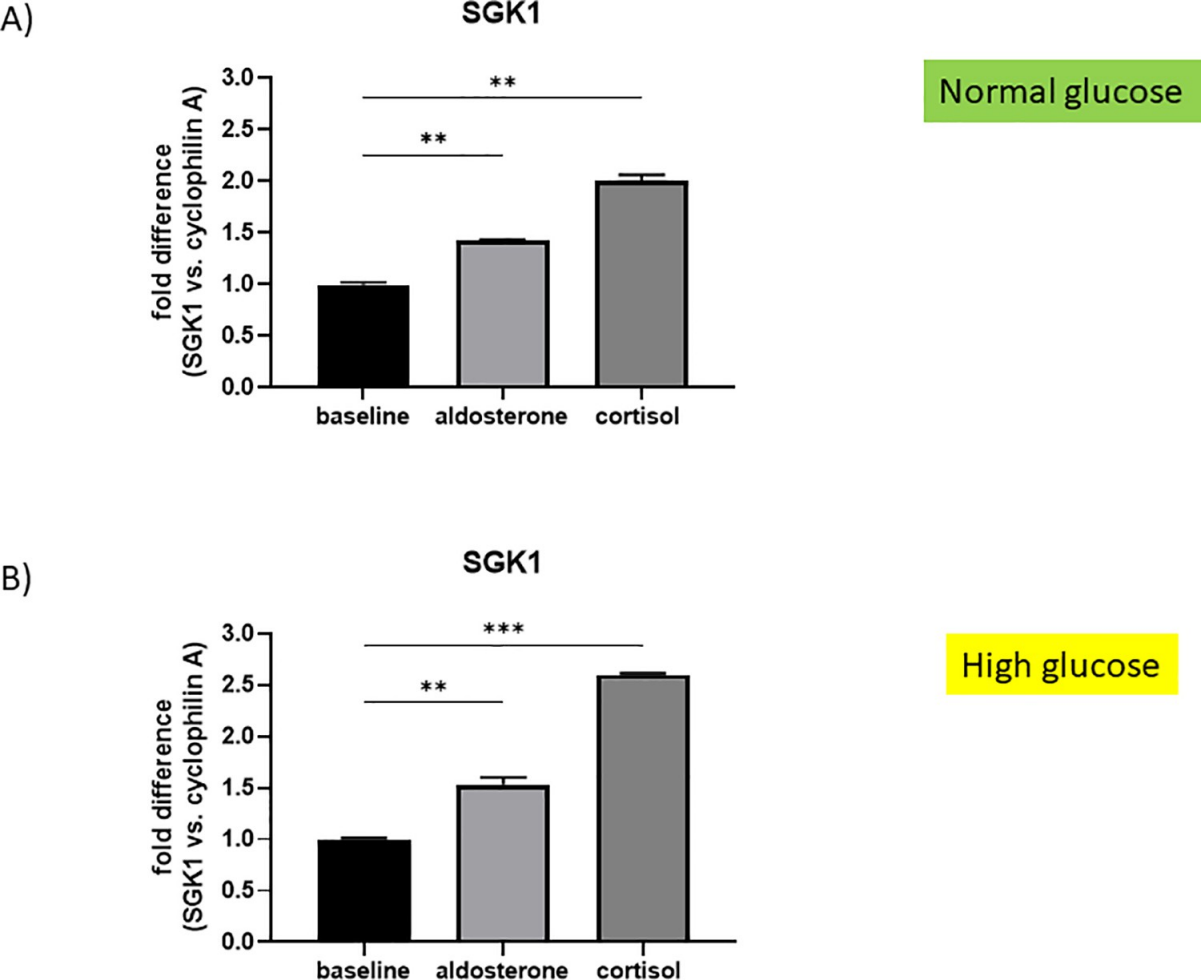
mRNA expression of SGK1 in HRMC in normal and high glucose conditions upon aldosterone and cortisol stimulation. A) mRNA expression of SKG1 in HRMC. Normal glucose conditions (5.25mM). Baseline control was EtOH. Fold difference is shown with cyclophilin A as endogenous control. Aldosterone (**p = 0.0026) and cortisol (**p = 0.0002) significantly stimulated SGK1 expression in HRMC on a normal glucose background. B) mRNA expression of SGK1 in HRMC. High glucose conditions (25.25mM). Baseline control was EtOH. Fold difference is shown with cyclophilin A as endogenous control. Aldosterone (**p = 0.0018) and cortisol (***p<0.0001) significantly stimulated SGK1 expression in HRMC on a high glucose background. *p<0.05, **p<0.01, ***p<0.0001, ns = not significant.

HRMC were cultured with aldosterone or cortisol either in normal or high glucose conditions as described above. RNA was isolated and real-time PCR was performed to detect mRNA levels of SGK1. Both steroid hormones stimulated SGK1 expression significantly in normal and high glucose conditions. Normal glucose: aldosterone (**p = 0.0026, fold difference 1.43), cortisol (**p = 0.0002, fold difference 1.99) (Fig 6A); high glucose: aldosterone (**p = 0.0018, fold difference 1.53), cortisol (***p<0.0001, fold difference 2.60) (Fig 6B).

#### mRNA expression of TGFB1, FN1 and COL1A1 in HRMC stimulated with aldosterone and cortisol and the MR and GR inhibitors spironolactone and RU486 in high glucose conditions

**Fig 7:**
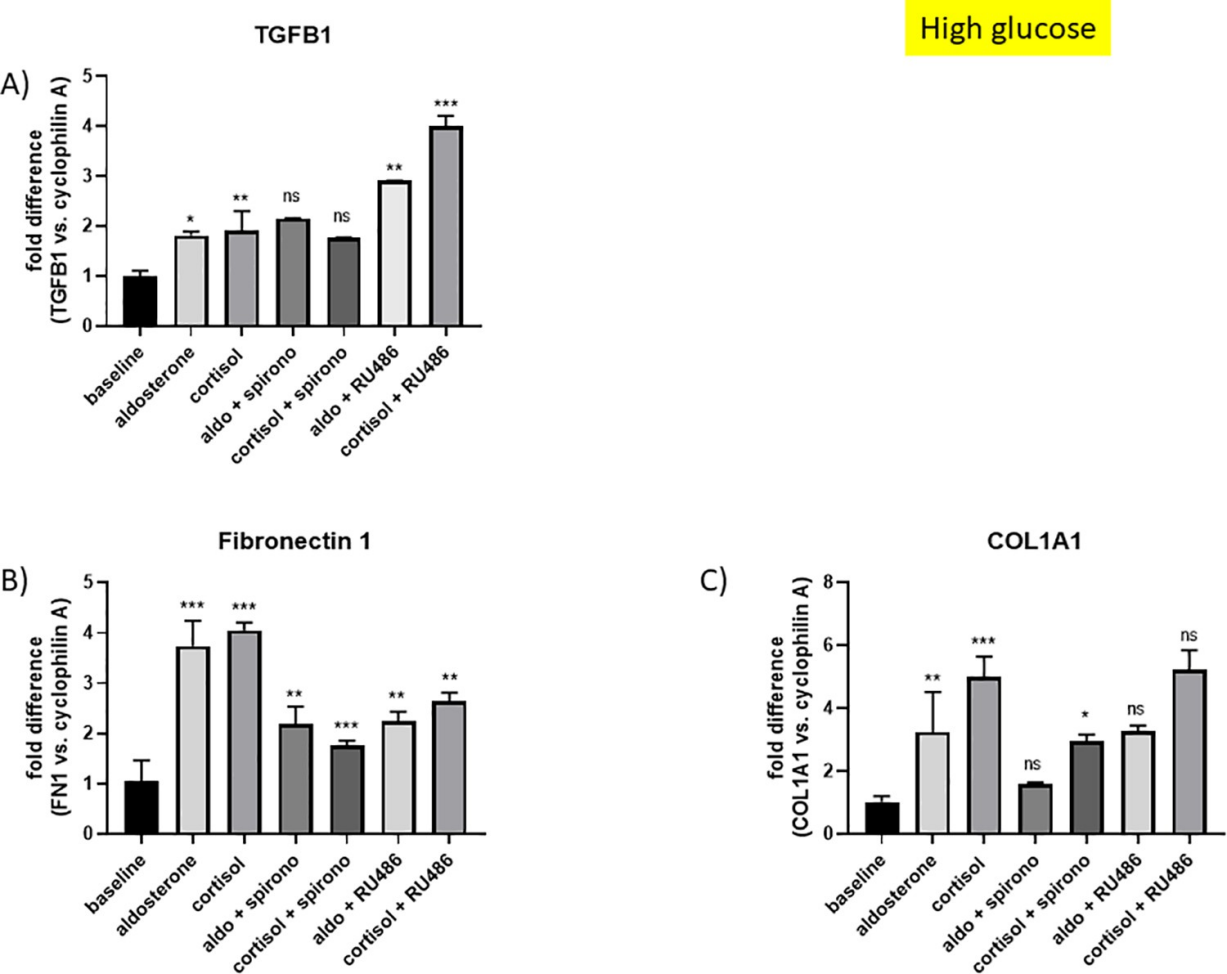
mRNA expression of TGFB1, FN1 and COL1A1 in HRMC upon aldosterone and cortisol stimulation with and without MR and GR inhibition in high glucose conditions. A) mRNA expression of TGFB1 in HRMC. Baseline was EtOH. Fold difference is shown with cyclophilin A as endogenous control. Aldosterone (*p = 0.015) and cortisol (**p = 0.004) significantly upregulated TGFB1 mRNA expression. The upregulation of TGFB1 by aldosterone and cortisol could not be changed with spironolactone (Aldo vs. Aldo + spironolactone: ns; cortisol vs. cortisol + spironolactone: ns). The combination Aldo + RU486 (**p = 0.002) and cortisol + RU486 (***p<0.0001) stimulated TGFB1 expression more than Aldo and cortisol alone. B) mRNA expression of FN1 in HRMC. Baseline was EtOH. Fold difference is shown with cyclophilin A as endogenous control. Aldosterone (***p<0.0001) and cortisol (***p<0.0001) significantly upregulated FN1 mRNA expression up to 4-fold. The upregulation of FN1 by aldosterone and cortisol could significantly be reduced by spironolactone (Aldo vs. Aldo + spironolactone: **p = 0.002; cortisol vs. cortisol + spironolactone: ***p<0.0001). RU486 was also able to reduce the upregulation of FN1 by aldosterone and cortisol. Aldo vs. Aldo + RU486 (**p = 0.002) and cortisol vs. cortisol + RU486 (**p = 0.004). C) mRNA expression of COL1A1 in HRMC. Baseline was EtOH. Fold difference is shown with cyclophilin A as endogenous control. Aldosterone (**p = 0.006) and cortisol (***p<0.0001) significantly upregulated COL1A1 mRNA expression. The upregulation of COL1A1 by aldosterone and cortisol could be reduced by spironolactone, although only significant in combination with cortisol (Aldo vs. Aldo + spironolactone: ns; cortisol vs. cortisol + spironolactone: *p = 0.023). RU486 was not able to reduce the upregulation of COL1A1 by aldosterone and cortisol. Aldo vs. Aldo + RU486 (ns) and cortisol vs. cortisol + RU486 (ns). ns = not significant. Mean +/- SD, One-way ANOVA, Dunnett’s multiple comparisons test, n = 3. *p<0.05, **p<0.01, ***p<0.0001, ns = not significant.

HRMC were cultured as described above. RNA was isolated and real-time PCR was performed to detect mRNA levels of TGFB1, FN1 and COL1A1. Aldosterone significantly stimulated TGFB1 (*p = 0.015, fold difference 1.81), FN1 (***p<0.0001, fold difference 3.74) and COL1A1 (**p = 0.006, fold difference 3.25) expression. Cortisol increased the expression of TGFB1 (**p = 0.004, fold difference 1.91), FN1 (***p<0.0001, fold difference 4.05) and COL1A1 (***p<0.0001, fold difference 5.0) likewise (Fig 7A–7C). Spironolactone, a MRA, was unable to reduce TGFB1 upregulation (aldosterone+spironolactone: p = 0.46, fold difference 2.16; cortisol+spironolactone: p = 0.97, fold difference 1.77) (Fig 7A), while it reduced stimulation of FN1 (aldosterone+spironolactone: **p = 0.002, fold difference 2.19; cortisol+spironolactone: ***p<0.0001, fold difference 1.76) and COL1A1 (aldosterone+spironolactone: p = 0.07, fold difference 1.59), cortisol+spironolactone: *p = 0.023, fold difference 2.95) (Fig 7B and 7C). The condition aldosterone + spironolactone did not reach significance in reducing COL1A1 expression. GR inhibition with RU486 led to an upregulation of TGFB1 by aldosterone and cortisol (aldosterone+RU486: **p = 0.002, fold difference 2.91; cortisol+RU486: ***p<0.0001, fold difference 4.01) (Fig 7A). RU486 reduced the aldosterone (aldosterone+RU486: **p = 0.002, fold difference 2.25) and cortisol (cortisol+RU486: **p = 0.004, fold difference 2.64) stimulated FN1 expression significantly (Fig 7B). COL1A1 upregulation by aldosterone and cortisol could not be reduced with RU486 (aldosterone+RU486: p>0.99, fold difference 3.29; cortisol+RU486: p = 0.99, fold difference 5.24) (Fig 7C).

#### mRNA expression of TGFB1, FN1 and COL1A1 in HRMC stimulated with aldosterone and cortisol in normal glucose conditions

HRMC were cultured as described above. RNA was isolated and real-time PCR was performed to detect mRNA levels of TGFB1, FN1 and COL1A1. Aldosterone significantly stimulated TGFB1 (***p<0.0001), FN1 (**p = 0.0005) and COL1A1 (*p = 0.023) expression. Cortisol increased the expression of FN1 (*p = 0.020) and COL1A1 (*p = 0.037), while it had no effect on TGFB1 expression (S5A–S5C Fig).

## Discussion

Comparing the urinary steroid hormone profile of patients with diabetic kidney disease to healthy controls revealed an upregulated mineralo- and glucocorticoid metabolism in the diabetic cohort. In response, higher apparent systemic HSD11B2 activity suggested less activation of the MR by cortisol in diabetic patients. Absence of glucocorticoid deactivation as HSD11B2 was missing in glomerular endothelial (HRGEC) and mesangial (HRMC) cells, allowing MR activation by cortisol in these cells as expression of MR, GR and serum- and glucocorticoid-regulated kinase 1 (SGK1), a down-stream signal of MR and GR activation, was unaltered in HRMC and HRGEC cultured in high glucose conditions. Consequently, Aldo and cortisol significantly upregulated SGK1 expression in HRMC both, in normal and in high glucose conditions, thus promoting an upregulated expression of pro-fibrotic genes in HRMC. High glucose conditions further increased the pro-fibrotic effect. Inhibitory experiments excluded a selective effect of the MR on the upregulation of these pro-fibrotic genes as the effect was also GR-dependent.

In detail, increased levels of urinary TH-Aldo in diabetic men and of 18-OH-THA in diabetic women could be discovered in our cohort; these could be markers of an increased aldosterone production as shown in other studies that found increased levels of plasma Aldo in type 2 diabetic patients [6, 45, 46]. Besides an increased aldosterone metabolism in diabetic patients, an increased glucocorticoid metabolism was found in our cohort reflected by elevated excretion of cortisol and cortisone metabolites. Baudrand et al. [47] found in line with our results, that increased urinary glucocorticoid metabolites were associated with metabolic syndrome, hypoadiponectinemia, insulin resistance and β-cell dysfunction. The study of Andrews et al. [48] showing increased cortisol metabolism in patients with glucose intolerance further strengthens our results.

Consistent with other studies [2–7] a normal apparent systemic CYP11B2 activity and a similar excretion of mineralocorticoids in diabetic patients compared to healthy controls could be found in women. Only in men, CYP11B2 activity was increased when calculated as THB/TH-Aldo ratio, but it was normal when calculated as ratio 18-OH-THA/TH-Aldo. Corticosterone (B), as reflected by THB in the urine is converted in a two-step process to Aldo, which explains the differential results in CYP11B2 activity in men. B is converted to 18-hydroxycorticosterone by the 18-hydroxylase (CMO I). 18-hydroxy-B is then in a rate limiting step further converted to Aldo by the 18-oxidase (CMO II). The calculated CYP11B2 activity depends on which CMO level the activity of the aldosterone synthase is estimated.

CYP11B1 enzyme activity was lower in diabetic men in line with a reduced cortisol excretion in this group. Diabetic and control women did not show a significant difference in CYP11B1 activity which was reflected in equal cortisol excretion among diabetic and healthy women. Overall, there persists an increased synthesis of glucocorticoid metabolites.

Diabetic men and women showed increased systemic HSD11B2 activity as compared to their healthy controls, suggesting, that cortisol inactivation is triggered in diabetic patients. It needs to be investigated if this change in MR selectivity is a rescue mechanism in order to inhibit cortisol from binding to the MR or the GR or if the resulting metabolites are functional and have a disease-modulating role. The group of Schnackenberg et al. has shown that chronic inhibition of HSD11B1 activity, meaning inhibiting the back-conversion of cortisone to cortisol, decreases hypertension, insulin resistance, and hypertriglyceridemia in metabolic syndrome [49], which points out that cortisol is a negative trigger in some conditions, especially in MR expressing tissues lacking HSD11B2. In contrast to our clinical results, Gant et al. [50] found no difference in the urinary excretion of the glucocorticoid metabolites 5β-THF, 5α-THF and THE in type 2 diabetes as compared to healthy subjects. This group furthermore found a higher cortisol/cortisone ratio in diabetic patients, which is contrary to our finding. We explain this discrepancy by a different type 2 diabetic cohort. Our cohort represents patients with chronic diabetic kidney disease, while the diabetic nephropathy patients of Gant et al. made up only 49% of the cohort. Furthermore, they allowed a maximum of one class of antihypertensive drugs for hypertensive patients during their study, while we excluded all of them before and during the urine sampling. This might additionally explain the different results.

Absent HSD11B2 expression in HRMC and HRGEC supports the hypothesis that Aldo and cortisol have both access to the MR in these two glomerular cell types and makes the local positive treatment effect of MRAs on these two glomerular cell types evident. In vivo studies confirmed activation of MR by glucocorticoids in settings where HSD11B2 was inhibited [51]. Even though the literature is controversial regarding the expression of HSD11B2 in glomerular cells [52–54] we are convinced of the absence of HSD11B2 in human glomerular endothelial and mesangial cells as assessed by real-time PCR. The reason for the controversy can be explained by the purity of the material. We worked with purchased, well characterized primary cells with high purity, while most other studies were performed either with podocytes or with whole glomeruli or kidney tissue which both reflect a mixture of cells.

HRMC and HRGEC were characterized as steroid hormone responsive cells, as they functionally express the MR and GR. Absence of HSD11B2 leads to MR activation by cortisol. Cortisol stimulation increased expression of pro-fibrotic genes. MR and GR are differentially involved in pro-fibrotic signaling, depending on which pro-fibrotic gene is investigated. The MRA spironolactone was unable to reduce TGFB1 upregulation by Aldo and cortisol, while it significantly reduced the upregulation of FN1 and COL1A1. GR inhibition with RU486 further stimulated Aldo and cortisol dependent upregulation of TGFB1. A similar observation was made in 2009 as researchers found that cortisol increased cardiac infarct size and that this effect was blocked by spironolactone, but not by RU486 [36]. GR inhibition with RU486 reduced the Aldo and cortisol stimulated FN1 expression significantly, suggesting GR involvement. COL1A1 upregulation by Aldo and cortisol could not be reduced with RU486, suggesting that this upregulation is not GR dependent. Overall the stimulation of the pro-fibrotic effects of Aldo and cortisol were more pronounced in high glucose conditions as compared to normal glucose conditions, suggesting that glucose itself provides an environment that favors progression of glomerular fibrosis. The used MRA spironolactone has besides a high anti-mineralocorticoid activity also a moderate anti-androgenic activity. The effects related to the partial inhibition of the androgen receptor were not assessed in this work. RU486 is not only a GR antagonist, it can also bind to the progesterone and androgen receptor. As its relative binding affinity at the GR is more than ten times that of cortisol and the assays were performed in steroid-free conditions with cortisol as exclusive ligand, the effects seen in HRMC are most likely based on GR inhibition. In further studies, involvement of other steroid hormone receptors should be investigated as well as the effects of other MR and GR ligands such as 11-deoxycorticosterone and dexamethasone.

The strength of this study is the thorough analysis of different urinary steroid hormone metabolites in well-characterized cohorts with high quality equipment using MS-based methods. Combining clinical and primary cell culture data now suggest that the MR expression remains stable in HRMC and HRGEC under high glucose conditions, but that the MR selectivity is changed and the MR activity is upregulated in HRMC under disease promoting conditions.

One limitation of our study is a limited number of diabetic patients in our cohort. These results need to be confirmed in a larger cohort and compared to plasma concentrations of steroids. Further studies for specific organ responses other than the kidney—especially for the heart—should be designed. Because there is a temporal relationship between steroid and protein excretion while glucocorticoids are partially bound to a binding protein, it remains uncertain whether proteinuria has an influence on the excretion of steroid hormone metabolites.

It is self-evident that any effect of MRAs onto two isolated cell lines cannot be extrapolated to systemic effects. In vivo studies correlating individual glomerular fibrosis with the steroid hormone profile of a patient should be performed.

To summarize, MR activation promotes pro-fibrotic signalling in mesangial cells, counteracted by MRAs. We strongly anticipate that the positive treatment effect of MRAs in diabetic kidney disease can be explained, at least in part, by these findings.

## Supporting information

S1 Fig Urinary excretion of tetrahydrodeoxycorticosterone (TH-DOC), tetrahydrocorticosterone (THB) and 18-OH-tetrahydrocorticosterone (18-OH-THA) in diabetic patients and healthy controls. Schematic overview of the mineralocorticoid metabolism in blood, tissue and urine of humans on the left. Urinary excretion of the mineralocorticoids TH-DOC, THB and 18-OH-THA in non-diabetic (n = 155 for men, n = 161 for women) and diabetic patients (n = 21 for men, n = 20 for women) measured by GC-MS. The groups were matched for age and gender. Diabetic and non-diabetic men and women excreted equal amounts of TH-DOC and of THB. Diabetic women excreted significantly more 18-OH-THA than healthy controls a finding absent in men. A) TH-DOC ns (men and women). B) THB ns (men and women). C) 18-OH-THA ns (men), ***p<0.0001 (women). Kolmogorov-Smirnov test (non-parametric, unpaired). Dot blot is shown with Log (10) scale and steroid concentrations are displayed in ug/24h. White circles = control men, black circles = diabetic men, white squares = control women, black squares = diabetic women.
(TIF)

S2 Fig Urinary excretion of 11-deoxycortisol (THS), cortisol and cortisone in diabetic patients and healthy controls. Urinary excretion of the glucocorticoids THS, cortisol and cortisone in non-diabetic (n = 155 for men and n = 161 for women) and diabetic patients (n = 21 for men and n = 20 for women) measured by GC-MS. The groups were matched for age and gender. Diabetic men and women excreted significantly more THS as their healthy controls. Cortisol excretion was significantly reduced in diabetic men, while there was no difference in cortisol excretion between diabetic and healthy women. Diabetic men excreted equal amounts of cortisone as healthy men, while diabetic women excreted significantly more cortisone as their controls. A) 11-deoxycortisol (THS) **p = 0.006 (men), **p = 0.006 (women). B) cortisol *p = 0.013 (men), ns (women). C) cortisone ns (men), **p = 0.009 (women). Kolmogorov-Smirnov test (non-parametric, unpaired). Dot blot is shown with Log (10) scale and steroid concentrations are displayed in ug/24h. White circles = control men, black circles = diabetic men, white squares = control women, black squares = diabetic women.
(TIF)

S3 Fig mRNA expression of MR and GR in HRMC and HRGEC upon high glucose stimulation. A) mRNA expression of MR in HRMC. Control was PBS. Fold difference is shown with cyclophilin A as endogenous control. High glucose levels (25.25mM) did not change MR expression. ns (p = 0.41). B) mRNA expression of GR in HRMC. Control was PBS. Fold difference is shown with cyclophilin A as endogenous control. High glucose levels (25.25mM) did not change GR expression. ns (p = 0.72). C) mRNA expression of MR in HRGEC. Control was PBS. Fold difference is shown with cyclophilin A as endogenous control. High glucose levels (25.25mM) did not change MR expression. ns (p = 0.18). D) mRNA expression of GR in HRGEC. Control was PBS. Fold difference is shown with cyclophilin A as endogenous control. High glucose levels (25.25mM) did not change GR expression. ns (p = 0.23). ns = not significant. Mean +/- SD, unpaired t test, n = 3.
(TIF)

S4 Fig mRNA expression of SGK1 in HRMC and HRGEC upon high glucose stimulation. A) mRNA expression of SGK1 in HRMC. Control was PBS. Fold difference is shown with cyclophilin A as endogenous control. High glucose levels (25.25mM) did not change SGK1 expression. ns (p = 0.80). B) mRNA expression of SGK1 in HRGEC. Control was PBS. Fold difference is shown with cyclophilin A as endogenous control. High glucose levels (25.25mM) did not change SGK1 expression. ns (p = 0.85). ns = not significant. Mean +/- SD, unpaired t test, n = 3.
(TIF)

S5 Fig mRNA expression of TGFB1, FN1 and COL1A1 in HRMC upon aldosterone and cortisol stimulation in normal glucose conditions. A) mRNA expression of TGFB1 in HRMC. Baseline was EtOH. Fold difference is shown with cyclophilin A as endogenous control. Aldosterone (***p<0.0001) significantly upregulated TGFB1 mRNA expression. Cortisol did not change TGFB1 expression (ns, p = 0.63). B) mRNA expression of FN1 in HRMC. Baseline was EtOH. Fold difference is shown with cyclophilin A as endogenous control. Aldosterone (**p = 0.0005) and cortisol (*p = 0.020) significantly upregulated FN1 mRNA expression. C) mRNA expression of COL1A1 in HRMC. Baseline was EtOH. Fold difference is shown with cyclophilin A as endogenous control. Aldosterone (*p = 0.023) and cortisol (*p = 0.037) significantly upregulated COL1A1 mRNA expression. ns = not significant. Mean +/- SD, One-way ANOVA, Dunnett’s multiple comparisons test, n = 3.
(TIF)

## Abbreviations

AT1R: angiotensin II receptor type 1
Aldo: aldosterone
Ang II: angiotensin II
COL1A1: Collagen type 1 alpha 1
CYP11B1: steroid 11 beta-hydroxylase
CYP11B2: aldosterone synthase
ECM: endothelial cell medium
E: cortisone
EtOH: ethanol
F: cortisol
Fibronectin 1: FN1
GR: glucocorticoid receptor
HEK293: human embryonic kidney cells
HPA: hypothalamic pituitary adrenal axis
HRGEC: human renal glomerular endothelial cells
HRMC: human renal mesangial cells
HSD11B1: 11beta-Hydroxysteroid dehydrogenase type 1
HSD11B2: 11beta-Hydroxysteroid dehydrogenase type 2
JEG-3: human choriocarcinoma cell line
MCM: mesangial cell medium
MR: mineralocorticoid receptor
MRAs: mineralocorticoid receptor antagonists
RAAS: renin-angiotensin-aldosterone system
SKIPOGH: Swiss Kidney Project on Genes in Hypertension
TGFB1: Transforming growth factor beta 1
TH-Aldo: tetrahydroaldosterone
18-OH-THA: 18-hydroxytetrahydrocorticosterone
THB: tetrahydrocorticosterone
TH-DOC: tetrahydrodeoxycorticosterone
THE: tetrahydrocortisone
5α-THF: 5α-tetrahydrocortisol
5β-THF: 5β-tetrahydrocortisol
THS: tetrahydrosubstance S
