# Supplementary figures and images for: Increased glucocorticoid metabolism in diabetic kidney disease
Source: PLoS One. 2022 Jun 24;17(6):e0269920. doi: 10.1371/journal.pone.0269920 (PMC9231809; doi:10.1371/journal.pone.0269920)

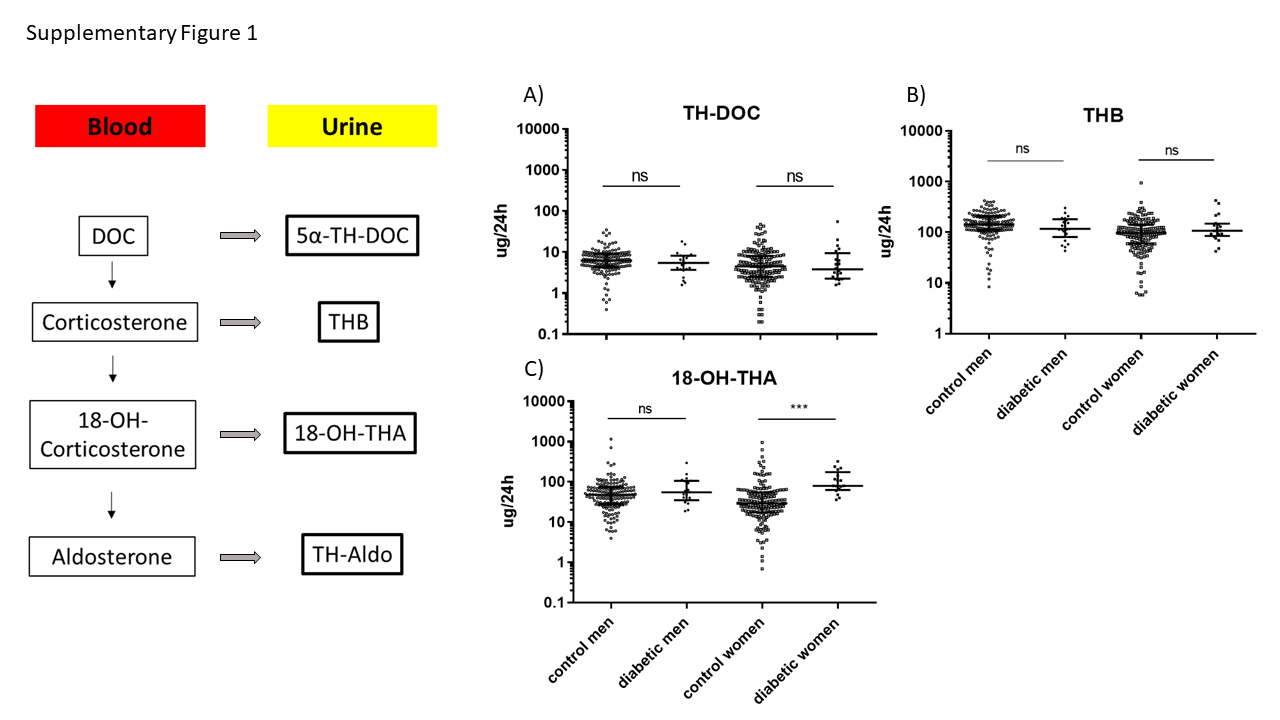

Supplement: S1 Fig — Schematic overview of the mineralocorticoid metabolism in blood, tissue and urine of humans on the left. Urinary excretion of the mineralocorticoids TH-DOC, THB and 18-OH-THA in non-diabetic (n = 155 for men, n = 161 for women) and diabetic patients (n = 21 for men, n = 20 for women) measured by GC-MS. The groups were matched for age and gender. Diabetic and non-diabetic men and women excreted equal amounts of TH-DOC and of THB. Diabetic women excreted significantly more 18-OH-THA than healthy controls a finding absent in men. A) TH-DOC ns (men and women). B) THB ns (men and women). C) 18-OH-THA ns (men), ***p<0.0001 (women). Kolmogorov-Smirnov test (non-parametric, unpaired). Dot blot is shown with Log (10) scale and steroid concentrations are displayed in ug/24h. White circles = control men, black circles = diabetic men, white squares = control women, black squares = diabetic women. (TIF) [file pone.0269920.s001.tif]

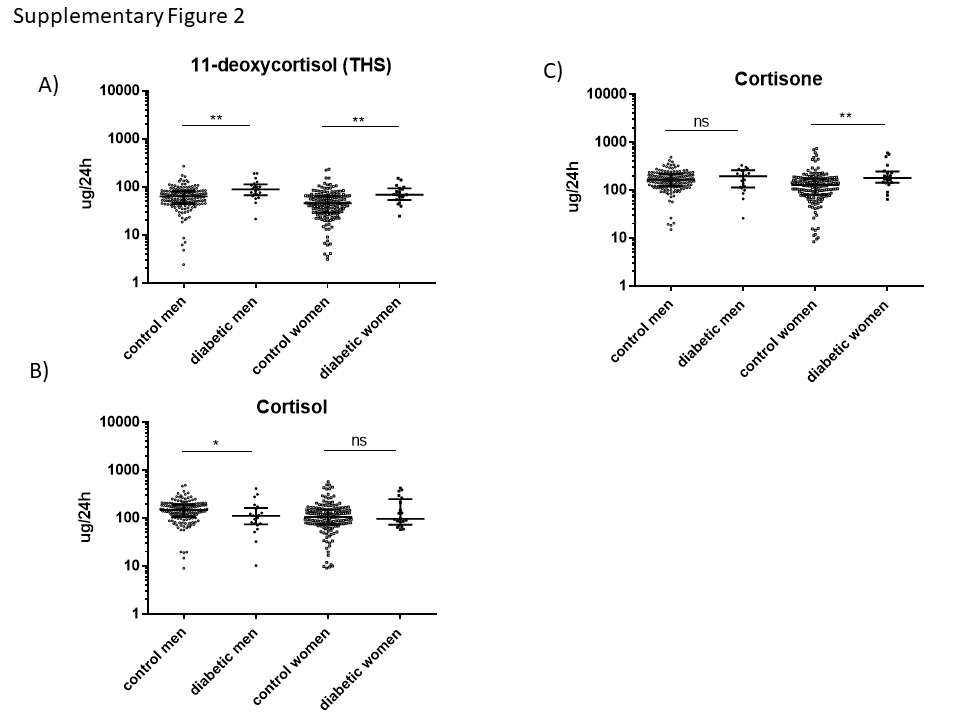

Supplement: S2 Fig — Urinary excretion of the glucocorticoids THS, cortisol and cortisone in non-diabetic (n = 155 for men and n = 161 for women) and diabetic patients (n = 21 for men and n = 20 for women) measured by GC-MS. The groups were matched for age and gender. Diabetic men and women excreted significantly more THS as their healthy controls. Cortisol excretion was significantly reduced in diabetic men, while there was no difference in cortisol excretion between diabetic and healthy women. Diabetic men excreted equal amounts of cortisone as healthy men, while diabetic women excreted significantly more cortisone as their controls. A) 11-deoxycortisol (THS) **p = 0.006 (men), **p = 0.006 (women). B) cortisol *p = 0.013 (men), ns (women). C) cortisone ns (men), **p = 0.009 (women). Kolmogorov-Smirnov test (non-parametric, unpaired). Dot blot is shown with Log (10) scale and steroid concentrations are displayed in ug/24h. White circles = control men, black circles = diabetic men, white squares = control women, black squares = diabetic women. (TIF) [file pone.0269920.s002.tif]

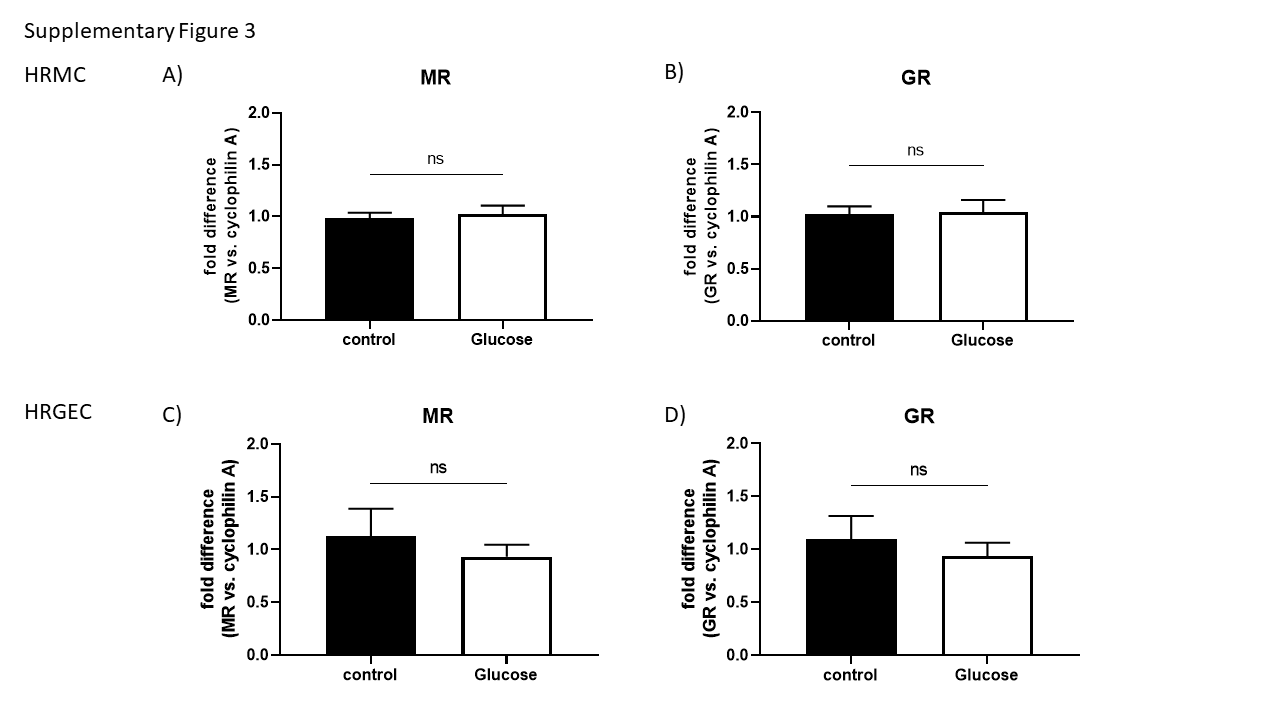

Supplement: S3 Fig — A) mRNA expression of MR in HRMC. Control was PBS. Fold difference is shown with cyclophilin A as endogenous control. High glucose levels (25.25mM) did not change MR expression. ns (p = 0.41). B) mRNA expression of GR in HRMC. Control was PBS. Fold difference is shown with cyclophilin A as endogenous control. High glucose levels (25.25mM) did not change GR expression. ns (p = 0.72). C) mRNA expression of MR in HRGEC. Control was PBS. Fold difference is shown with cyclophilin A as endogenous control. High glucose levels (25.25mM) did not change MR expression. ns (p = 0.18). D) mRNA expression of GR in HRGEC. Control was PBS. Fold difference is shown with cyclophilin A as endogenous control. High glucose levels (25.25mM) did not change GR expression. ns (p = 0.23). ns = not significant. Mean +/- SD, unpaired t test, n = 3. (TIF) [file pone.0269920.s003.tif]

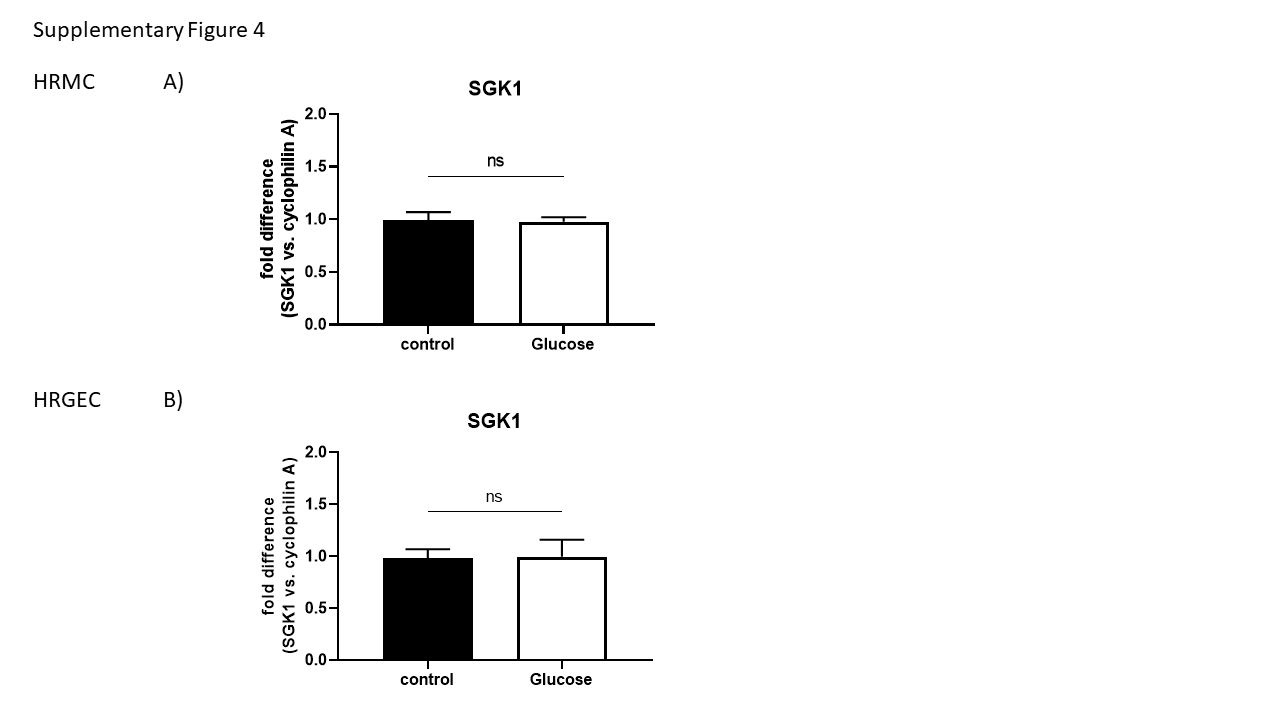

Supplement: S4 Fig — A) mRNA expression of SGK1 in HRMC. Control was PBS. Fold difference is shown with cyclophilin A as endogenous control. High glucose levels (25.25mM) did not change SGK1 expression. ns (p = 0.80). B) mRNA expression of SGK1 in HRGEC. Control was PBS. Fold difference is shown with cyclophilin A as endogenous control. High glucose levels (25.25mM) did not change SGK1 expression. ns (p = 0.85). ns = not significant. Mean +/- SD, unpaired t test, n = 3. (TIF) [file pone.0269920.s004.tif]

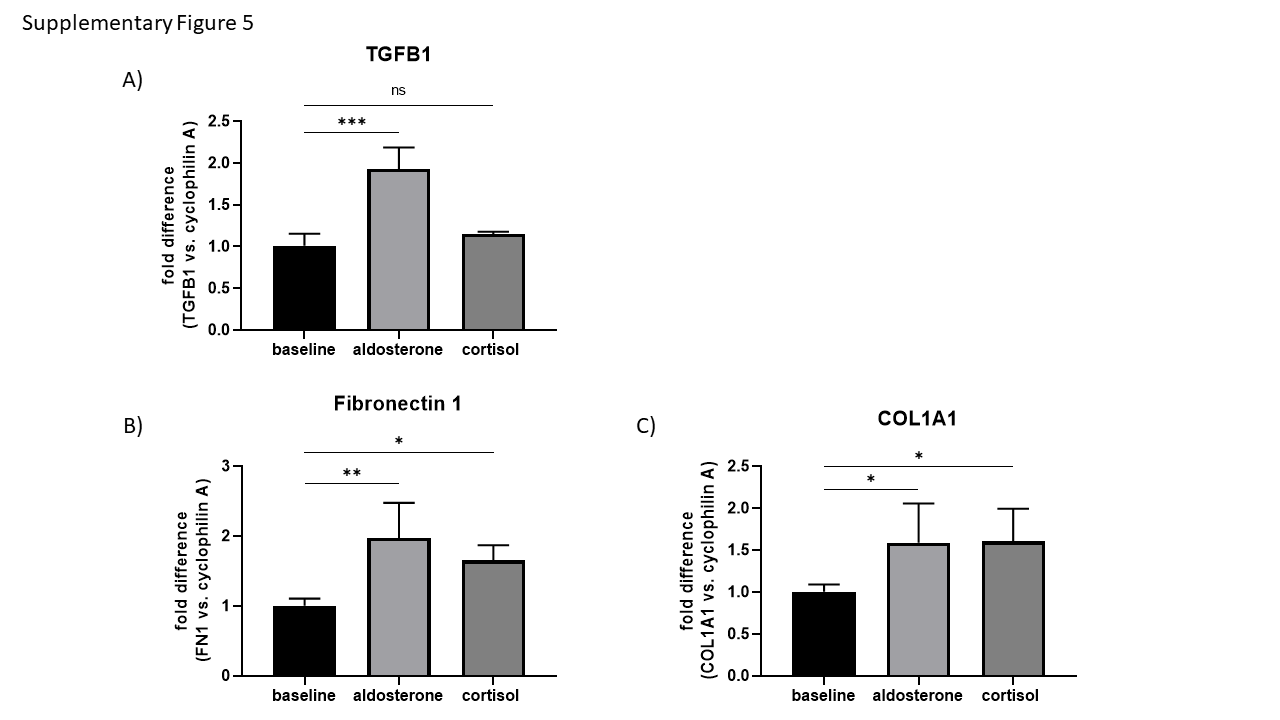

Supplement: S5 Fig — A) mRNA expression of TGFB1 in HRMC. Baseline was EtOH. Fold difference is shown with cyclophilin A as endogenous control. Aldosterone (***p<0.0001) significantly upregulated TGFB1 mRNA expression. Cortisol did not change TGFB1 expression (ns, p = 0.63). B) mRNA expression of FN1 in HRMC. Baseline was EtOH. Fold difference is shown with cyclophilin A as endogenous control. Aldosterone (**p = 0.0005) and cortisol (*p = 0.020) significantly upregulated FN1 mRNA expression. C) mRNA expression of COL1A1 in HRMC. Baseline was EtOH. Fold difference is shown with cyclophilin A as endogenous control. Aldosterone (*p = 0.023) and cortisol (*p = 0.037) significantly upregulated COL1A1 mRNA expression. ns = not significant. Mean +/- SD, One-way ANOVA, Dunnett’s multiple comparisons test, n = 3. (TIF) [file pone.0269920.s005.tif]
